# Supplementary material for: Mutational Diversity in the Quinolone Resistance-Determining Regions of Type-II Topoisomerases of Salmonella Serovars
Source: Antibiotics (Basel). 2021 Nov 26;10(12):1455. doi: 10.3390/antibiotics10121455 (PMC8698434; doi:10.3390/antibiotics10121455)
Supplement: Supplementary file 1 [file antibiotics-10-01455-s001.zip › Supplementary figures_QRDR review.pdf]

# Supplementary figures

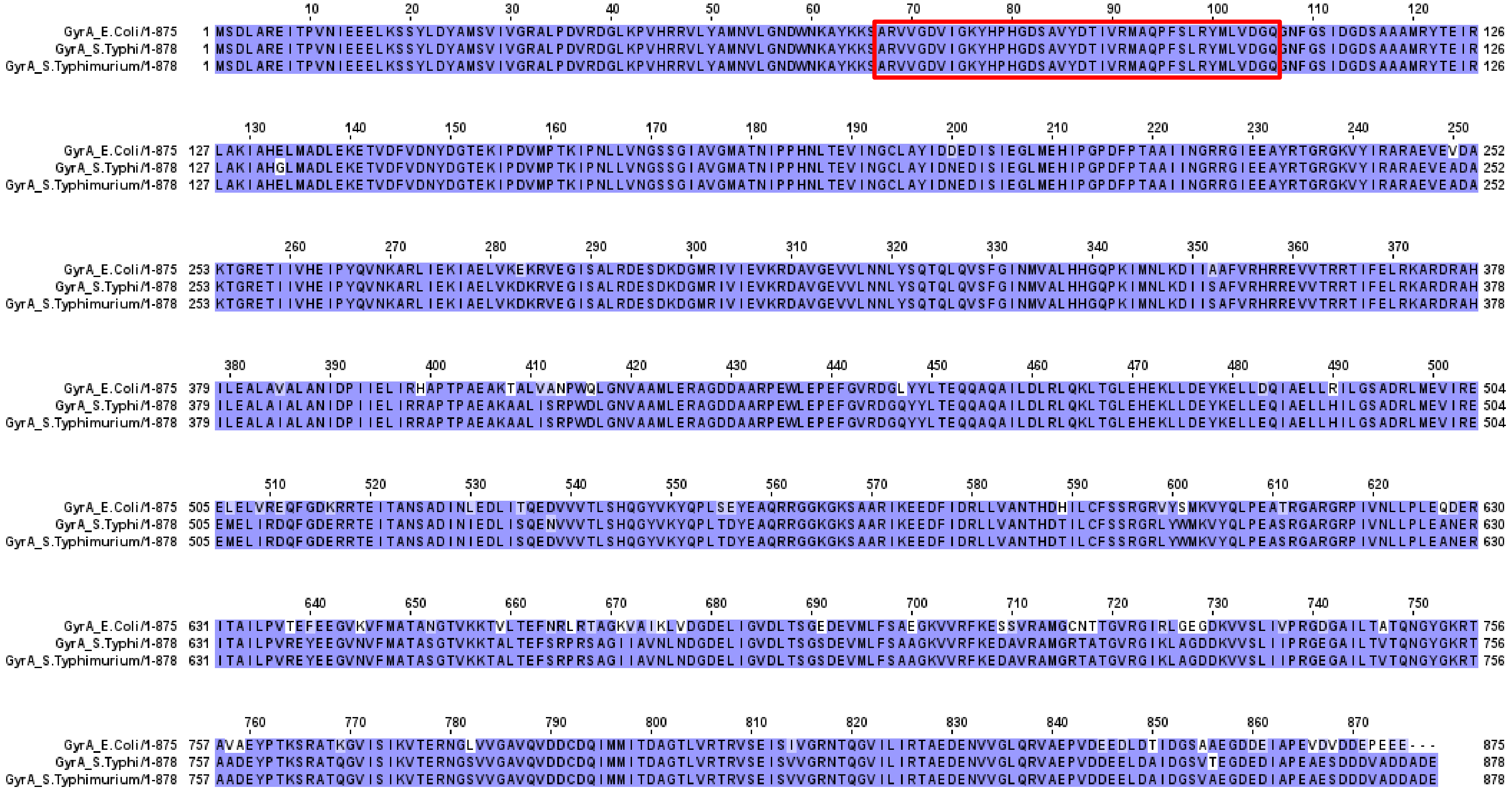


Figure S1: Amino acid sequence alignment of the GyrA homologs from *E. coli* (NCBI accession no. AAG57360.1), *S.* Typhi (NCBI accession no. AAO68297.1) and *S.* Typhimurium (NCBI accession no. AAL21173.1). The red box indicates the QRDR (Ala67-Gln106).

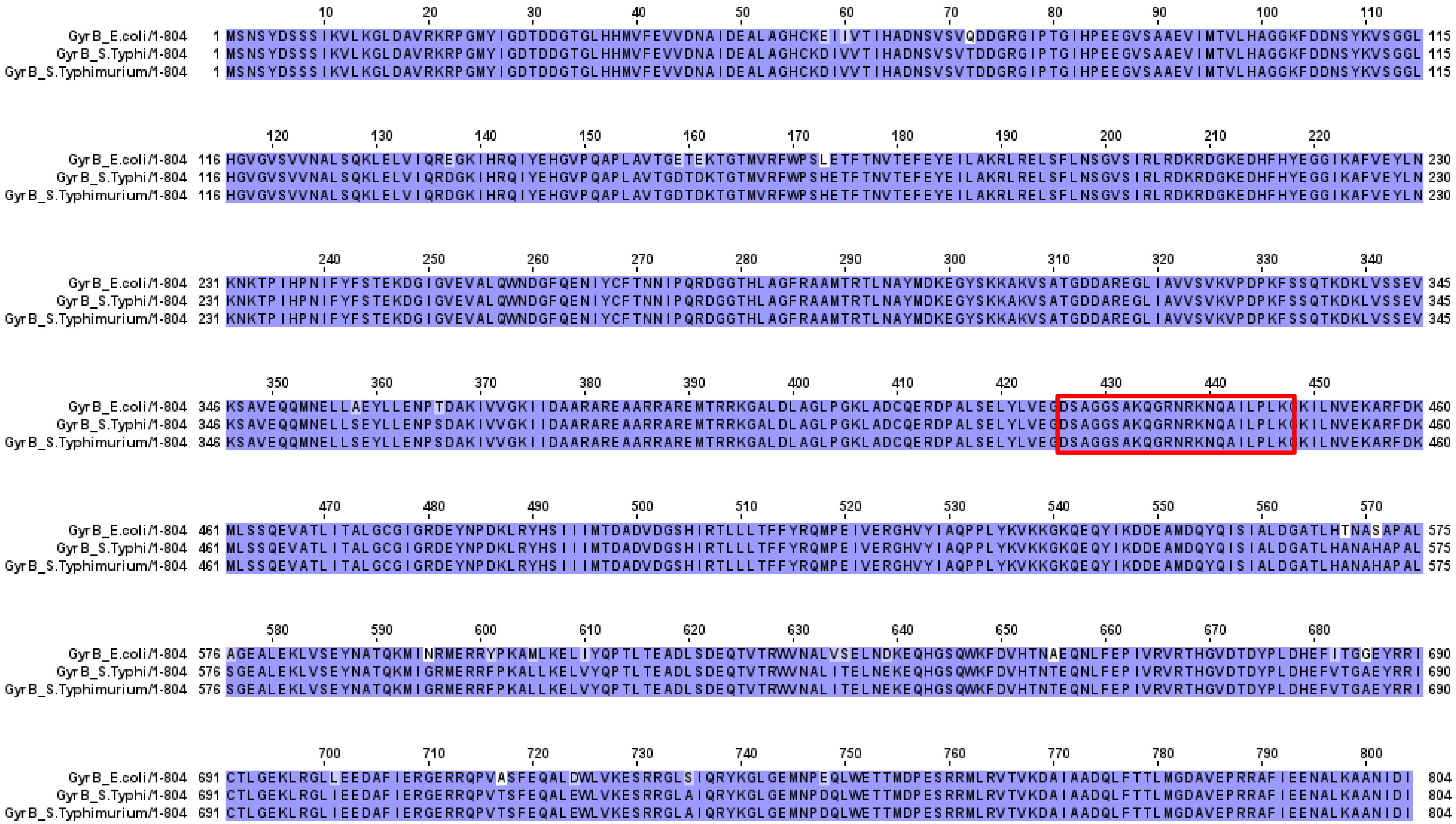

Figure S2: Amino acid sequence alignment of the GyrB homologs from *E. coli* (NCBI accession no. AAG58896.1), *S.* Typhi (NCBI accession no. AAO71180.1) and *S.* Typhimurium (NCBI accession no. AAL22694.1). The red box indicates the QRDR (Asp426-Lys447).

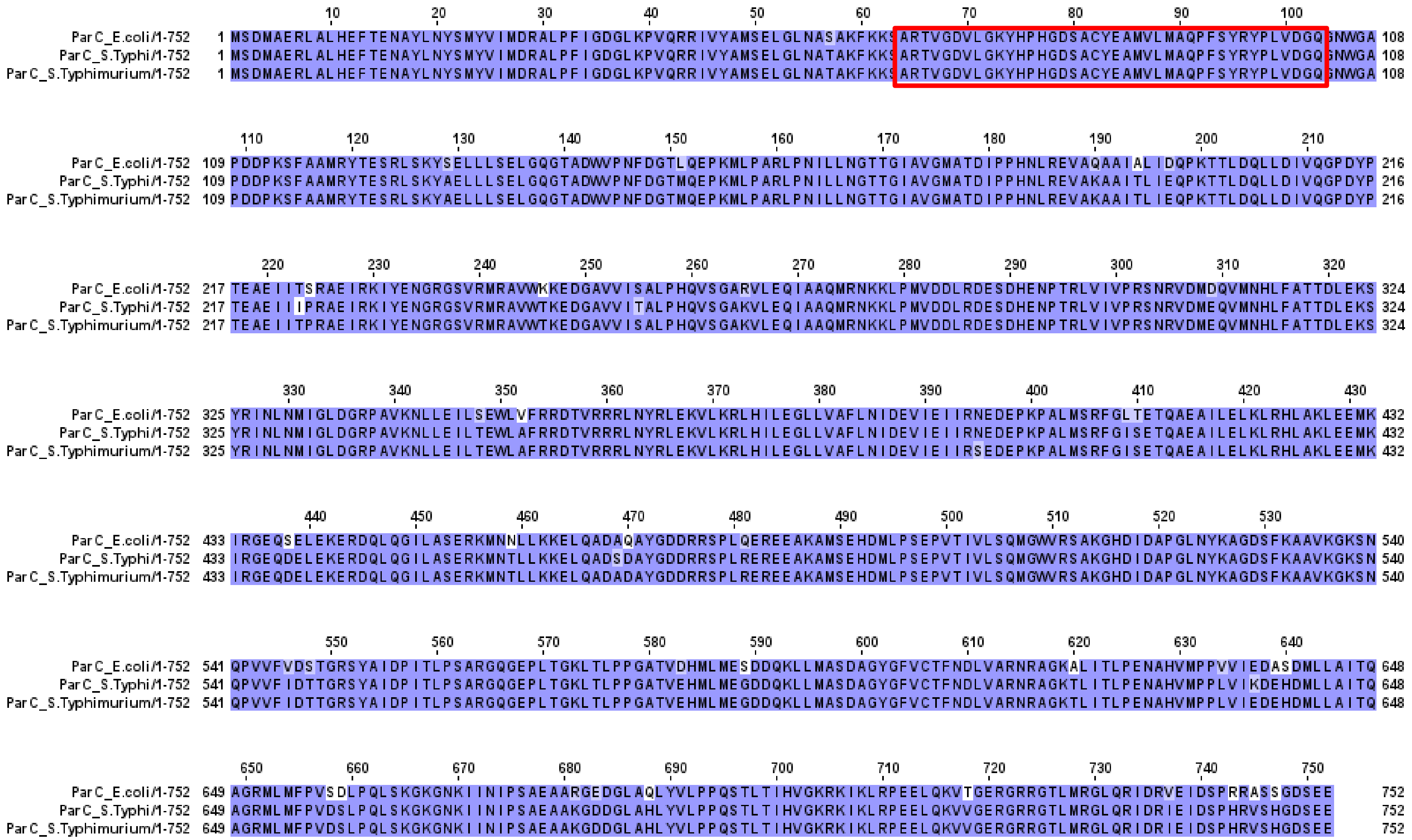


Figure S3: Amino acid sequence alignment of the ParC homologs from *E. coli* (NCBI accession no. AAG58155.1), *S.* Typhi (NCBI accession no. AAO70639.1) and *S.* Typhimurium (NCBI accession no. AAL22048.1). The red box indicates the QRDR (Ala64 to Gln103).

```
                                     10        20        30        40        50        60        70        80
ParE_E.coli/1-630              1 MTQTYNADAIEVLTGLEPVRRRPGMYTDTTRPNHLGQEVIDNSVDEALAGHAKRVDVILHADQSLEVIDDGRGMPVDIHPEEGVPAVELI  90
ParE_S.Typhi/1-630             1 MTQTYNADAIEVLTGLEPVRRRPGMYTDTTRPNHLGQEVIDNSVDEALAGHAKRVDVILHADQSLEVIDDGRGMPVDIHPEEGVPAVELI  90
ParE_S.Typhimurium/1-630       1 MTQTYNADAIEVLTGLEPVRRRPGMYTDTTRPNHLGQEVIDNSVDEALAGHAKRVDVILHADQSLEVIDDGRGMPVDIHPEEGVPAVELI  90

                                    100       110       120       130       140       150       160       170
ParE_E.coli/1-630             91 LCRLHAGGKFSNKNYQFSGGLHGVGISVVNALSKRVEXNVRRDGQVYNIAFENGEKVQDLQVVGNCGKRNTGTSVHFWPDETFFDSPRFS 180
ParE_S.Typhi/1-630            91 LCRLHAGGKFSNKNYQFSGGLHGVGISVVNALSKRVEVTVRRDGQVYNIAFENGEKVQDLQVVGTCGKRNTGTSVHFWPDESFFDSPRFS 180
ParE_S.Typhimurium/1-630      91 LCRLHAGGKFSNKNYQFSGGLHGVGISVVNALSKRVEVTVRRDGQVYNIAFENGEKVQDLQVVGTCGKRNTGTSVHFWPDESFFDSPRFS 180

                                    190       200       210       220       230       240       250       260
ParE_E.coli/1-630            181 VSRLTHVLKAKAVLCPGVEITFKDEINNTEQRWCYQDGLNDYLAEAVNGLPTLPEKPFIGNFAGDTEAVDWALLWLPEGGELLTESYVNL 270
ParE_S.Typhi/1-630           181 VSRLMHVLKAKAVLCPGVEITFKDEVNNSEQRWCYQDGLNDYLGEAVNGLPTLPEKPFIGNFNGETEAVDWALLWLPEGGELLTESYVNL 270
ParE_S.Typhimurium/1-630     181 VSRLMHVLKAKAVLCPGVEITFKDEVNNSEQRWCYQDGLNDYLGEAVNGLPTLPEKPFIGNFNGETEAVDWALLWLPEGGELLTESYVNL 270

                                    280       290       300       310       320       330       340       350
ParE_E.coli/1-630            271 IPTMQGGTHVNGLRQGLLDAMREFCEYRNILPRGVKLSAEDIWDRCAYVLSVKMQDPQFAGQTKERLSSRQCAAFVSGVVKDAFILWLNQ 360
ParE_S.Typhi/1-630           271 IPTMQGGTHVNGLRQGLLDAMREFCEYRNILPRGVKLSAEDIWDRCAYVLSVKMQDPQFAGQTKERLSSRQCAAFVSGVVKDAFSLWLNQ 360
ParE_S.Typhimurium/1-630     271 IPTMQGGTHVNGLRQGLLDAMREFCEYRNILPRGVKLSAEDIWDRCAYVLSVKMQDPQFAGQTKERLSSRQCAAFVSGVVKDAFSLWLNQ 360

                                    370       380       390       400       410       420       430       440
ParE_E.coli/1-630            361 NVQAAELLAEMAISSAQRRMRAAKKVVRKKLTSGPALPGKLADCTAQDLNRTELFLVEGDSAGGSAKQARDREYQAIMPLKGKILNTWEV 450
ParE_S.Typhi/1-630           361 NVQAAEQLAEMAIASAQRRLRAAKKVVRKKLTSGPALPGKLADCTAQDLNRTELFLVEGDSAGGSAKQARDREYQAIMPLKGKILNTWEV 450
ParE_S.Typhimurium/1-630     361 NVQAAEQLAEMAIASAQRRLRAAKKVVRKKLTSGPALPGKLADCTAQDLNRTELFLVEGDSAGGSAKQARDREYQAIMPLKGKILNTWEV 450

                                    460       470       480       490       500       510       520       530
ParE_E.coli/1-630            451 SSDEVLASQEVHDISVAIGIDPDSDDLSQLRYGKICILADADSDGLHIATLLCALFVKHFRALVKHGHVYVALPPLYRIDLGKEVYYALT 540
ParE_S.Typhi/1-630           451 SSDEVLASQEVHDISVAIGIDPDSDDLSQLRYGKICILADADSDGLHIATLLCALFVRHFRALVKNGHVYVALPPLYRIDLGKEVYYALT 540
ParE_S.Typhimurium/1-630     451 SSDEVLASQEVHDISVAIGIDPDSDDLSQLRYGKICILADADSDGLHIATLLCALFVRHFRALVKNGHVYVALPPLYRIDLGKEVYYALT 540

                                    550       560       570       580       590       600       610       620
ParE_E.coli/1-630            541 EEEKEGVLEQLKRKKGKPNVQRFKGLGEMNPMQLRETTLDPNTRRLVQLTIDDEDDQRTDAMMDMLLAKKRSEDRRNWLQEKGDMAEIEV 630
ParE_S.Typhi/1-630           541 EEEKAGVLEQLKRKKGKPNVQRFKGLGEMNPMQLRETTLDPNTRRLVQLTISDEDDQRTNAMMDMLLAKKRSEDRRNWLQEKGDLADLDV 630
ParE_S.Typhimurium/1-630     541 EEEKAGVLEQLKRKKGKPNVQRFKGLGEMNPMQLRETTLDPNTRRLVQLTISDEDDQRTNAMMDMLLAKKRSEDRRNWLQEKGDLADLDV 630
```

Figure S4: Amino acid sequence alignment fofor the ParE homologs from *E. coli* (NCBI accession no. AAG58169.1), *S.* Typhi (NCBI accession no. AAO70645.1) and *S.* Typhimurium (NCBI accession no. AAL22055.1). The red box indicates the QRDR (Asp420-Lys441).
