# Supplementary material for: Mutational Diversity in the Quinolone Resistance-Determining Regions of Type-II Topoisomerases of Salmonella Serovars
Source: Antibiotics (Basel). 2021 Nov 26;10(12):1455. doi: 10.3390/antibiotics10121455 (PMC8698434; doi:10.3390/antibiotics10121455)
Supplement: Supplementary file 1 [file antibiotics-10-01455-s001.zip › Supplementary tables.pdf]

Table S1: Typhoidal *Salmonella*

Serovar	CIP^r	GyrA	GyrB	ParC	ParE	PMQR	Efflux	MIC (mg/L)	Reference
S. Typhi	S	Asp82Asn	-	-	-	-	ND	0.03	[76]
	I	-	-	Thr57Ser	-	-	ND	0.125	[76]
	R	Ser83Phe	-	Thr57Ser	-	-	No	1	[87]
	R	Ser83Tyr	-	Thr57Ser	-	-	No	2	[87]
	I-R	Ser83Tyr	-	Gly72Ser	-	-	No	0.25-0.38	[78]
	I – R	Ser83Phe	-	-	-	-	No/ND	0.19-6	[23, 34, 72, 74, 75, 76, 78, 85, 87, 100]
	R	Ser83Phe	-	-	-	*qnrB2*	ND	1	[79]
	R	Ser83Phe	-	-	-	*qnrS1*	ND	1-2	[102]
	R	Ser83Phe	-	Glu84Lys	ND	*qnrB*	No	>32	[78]
		Ser83Phe		Glu84Lys	ND	-	No	0.38->32	[78]
	I	Ser83Leu	-	-	-	-	ND	0.38-0.5	[34]
	R	Ser83Leu Asp87Asn	-	Ser80Ile	-		Yes	4	[87]
	I	Ser83Tyr	-	-	-	-	No/ND	0.25-0.5	[34, 72, 74, 78, 85, 87, 95]
	R	Ser83Tyr	-	-	-	*qnrS1*	Yes	4	[82, 85]
	S – I	Asp87Asn	-	-	-	-	No/ND	0.06-0.5	[72, 74, 85, 95]
	S – I	Asp87Gly	-	-	-	-	ND	0.06-0.5	[72]
	R	Asp87Tyr	-	-	-	*qnrS1*	ND	3	[34]
	S	Ser83Phe	-	-	Asp420Asn	-	ND	0.06	[72]
	I	Ser83Tyr	-	-	Ser493Phe	-	ND	0.38	[76]
	R	Ser83Phe Asp87Val	-	Ser80Ile	-	-	ND	>32	[34, 86]
	I – R	Ser83Phe Asp87Asn	-	-	-	-	ND	0.25-4	[74, 96]
	R	Ser83Phe Asp87Asn	-	Ser80Ile	-	-	No/ND	0.5- 63	[72, 74, 76, 78, 80, 87]
	R	Ser83Phe	-	Ser80Ile	-	-	No	2	[87]
	S	Glu133Gly	-	-	-	-	ND	0.06	[89, 90, 95]
	S	Glu133Gly	Ser426Gly	-	-	-	ND	NG	[95]
	R	Ser83Phe Asp87Asn	-	Thr57Ser Glu84Lys	-	-	ND	NG	[95]
	S	Ser83Phe Glu133Gly	-	Asp79Gly	-	-	ND	NG	[95]
	S	Ser83Phe Glu133Gly	-	-	Glu543Lys	-	ND	NG	[95]

	S	Ser83Phe Glu133Gly	-	-	Val560Gly	-	ND	NG	[95]
	I	Ser83Phe Glu133Gly	-	-	Ile444Ser	-	ND	NG	[95]
	S	Ser83Phe Glu133Gly	-	-	Tyr434Ser	-	ND	NG	[95]
	R	Ser83Phe Asp87Gly	-	Glu92Lys	-	-	Yes	8-16	[85]
	I	Ser83Phe Glu133Gly	-	-	-	-	ND	0.25-0.38	[32, 90]
	R	Ser83Tyr Glu133Gly	-	-	-	*qnrS1*	ND	2	[34]
	I	Ser83Phe Glu133Gly	Ala574Val	-	-	-	ND	0.38	[34]
	I	Glu133GlySer83Phe	-	-	-	-	ND	0.5	[99]
	I	†Gly133Glu Ser83Phe	-	-	-	-	ND	≤1	[91]
	I – R	Ser83Phe	-	Trp106Gly	-	ND	ND	0.125-1	[96]
	R	Ser83Phe Asp87Asn Glu133Gly	-	Ser80Ile	-	ND	ND	7mm	[34]
	R	Ser83Phe Asp87Asn	Gly435Glu	Ser80Ile	-	-	ND	12	[76]
	I	Ser83Phe	Gly435Ala	-	-	-	ND	0.15	[76]
	I	Asp87Asn	Gly435Glu	-	-	-	ND	0.19	[76]
	I	Ser83Phe	Gly435Glu	-	-	-	ND	0.25	[76]
	I	Ser83Phe	Gly435Val	-	-	-	ND	0.5	[76]
	I	-	Ser464Tyr	-	-	-	ND	0.125	[72, 93]
	I	-	Ser464Phe	-	-	-	ND	0.125	[72, 93]
	S		Ser464Thr		-	-	ND	0.06	[93]
	I	Glu133Gly	Ser464Phe	-	-	-	ND	0.25	[99]
	S	-	Gln465Leu	-	-	-	ND	0.04-0.08	[72]
	S	-	Glu466Asp	-	-	-	ND	0.04	[72]
	S	-	Ala468Glu	-	-	-	ND	0.03	[72]
S. Paratyphi A	I	Ser83Tyr	-	-	-	-	ND	21mm	[34, 71]
	R	Ser83Phe Asp87Gly	ND	ND	-	-	ND	32	[100]
	R	Ser83Phe Asp87Asn	-	Thr57Ser Glu84Lys	-	-	ND	NG	[95]
		Ser83Phe			Ile444Ser				[95]
	I – R	Ser83Phe	-		-	-	ND	0.125-1	[71, 95]
	I	Ser83Phe	-	Thr57Ser	-	-	ND	0.5	[99]
	I	Ser83Phe	-	Thr57Ser	-	-	ND	21-26 mm	[34, 95]
	R – I	Asp87Asn	-	-	-	-	ND	0.125-1	[71]
	I	Asp87Tyr	-	Thr57Ser	-	ND	ND	22 mm	[34]
rat yp	I	Ser83Phe	-	-	-	-	ND	0.25-0.5	[34]
		Ser83Phe	-	-	Ser498Thr		ND		[95]

	I	Ser83Tyr	-	-	-	-	ND	28mm / ≤1	[34] [91]
	I	Asp87Tyr	-	-	-	-	ND	21-28mm	[34]
	I	Asp87Asn	ND	ND	ND	ND	ND	≤ 1	[91]
	I	Asp87Gly	-	-	-	-	ND	22-26mm / ≤1	[34] [91]
	I	Arg47Ser Asp87Gly	ND	ND	ND	ND	ND	≤ 1	[91]
S. Para-typhi C	I	Asp87Tyr	ND	ND	ND	ND	ND	≤ 1	[91]
	I	Asp87Gly	ND	ND	ND	ND	ND	≤ 1	[91]
	I	Asp87Asn	ND	ND	ND	ND	ND	≤ 1	[91]
	I	Ser83Phe Asp147Gly	ND	ND	ND	ND	ND	≤ 1	[91]

Table S1: Mutations in the QRDRs of typhoidal *Salmonella*, namely *Salmonella* Typhi, *Salmonella* Paratyphi A, B and C. Clinical and Laboratory Standards Institute (CLSI) guidelines [58] were followed to ensure a uniform classification of isolates showing ciprofloxacin resistance. According to these guidelines, resistant isolates have an MIC of 1–2 mg/L, isolates with intermediate susceptibility have an MIC of 0.12–0.5 mg/L, and susceptible isolates have an MIC of ≤ 0.06 mg/L. †Walther-Rausmussen and Hoiby [88] reported that *S.* Typhi has Gly instead of Glu at position 133 and the double mutant was Gly133Glu and Ser83Phe. CIP^r: ciprofloxacin resistance phenotype; ND: not determined; NG: not given; S: susceptible; I: intermediate susceptibility; R: resistant.

Table S2: *Salmonella* Enteritidis								
**CIP^r**	***gyrA***	***gyrB***	***parC***	***ParE***	**PMQR**	**Efflux**	**MIC (mg/L)**	**References**
I-R	Asp87Tyr	-	-	-	-	ND	0.09-1	[28, 34, 89, 106, 107, 108, 109, 110, 111, 113, 114, 115]
I-R	Asp87Gly	-	-	-	-	ND	0.125-1	[34, 89, 107, 108, 111, 112, 114, 115, 116]
I	Asp87Tyr	-	-	-	*qnrB*	ND	0.086	[113]
R	Asp87Tyr	-			*qnrS2*	ND	2	[28]
R	Asp87Tyr	-	-	-	*qnrS1*	ND	1	[108]
I	Asp87Asn	-	-	-	-	ND	0.032-0.25	[89, 91, 107, 110, 113, 115, 116, 117]
R	Asp87Asn	-	-	-	*qnrS1*	ND	1	[108, 112]
R	Asp87Asn	-	-	-	*aac(6')-lb-cr*	ND	2	[32]
I	Ser83Ile	-	-	-	-	ND	0.09-0.38	[108, 115]
I-R	Ser83Phe	-	-	-	-	ND	0.125-1	[28, 34, 106, 107, 108, 112, 113, 115, 116]
I	Ser83Phe	-	-	-	*qnrB*	ND	0.25	[32]
I	-	-	-	Val521Phe	*qnrS1*	ND	0.09	[108]
I	Ser83Phe	-	Thr57Ser	-	-	ND	0.25	[32]
I	Ser83Tyr	-	-	-	-	ND	0.25-0.5	[28, 34, 107, 108, 112, 115]
R	Ser83Tyr	-	-	-	*qnrS1*	ND	1-3	[34, 108, 115]
I	Ser83Tyr Asp87Gly	-	-	-	-	ND	0.125	[112]
I	Ser83Tyr Asp87Asn	-	-	-	-	ND	0.125-0.25	[34, 112]
S-I	Ser83Phe Asp87Asn	-	-	-	-	ND	0.064-0.125	[34, 112]
I	Ser83Phe Asp87Gly	-	-	-	-	ND	0.125	[112]
I	Ser83Phe Asp87Tyr	-	-	-	-	ND	4	[112, 115, 119]
R	Ser83Phe Asp87Ser	-	Ser67Cys Arg76Cys Cys80Arg	ND	-	Yes	256	[39]
I	Asp87Tyr Ala131Gly	-	-	-	-	ND	0.125	[112]
S	Ser97Pro	-				ND	0.06	[113]

Table S2: Mutations in the QRDRs of *Salmonella* Enteritidis. CLSI guidelines [58] were followed to ensure a uniform classification of isolates showing ciprofloxacin resistance. According to these guidelines, resistant isolates have an MIC of 1–2 mg/L, isolates with intermediate susceptibility have an MIC of 0.12–0.5 mg/L, and susceptible isolates have an MIC of ≤ 0.06 mg/L. Asp87Tyr substitution is predominant among *S.* Enteritidis isolates worldwide. PMQR determinants are rare, but present in isolates from Asia, Europe and America. The contribution of efflux to resistance has not been studied extensively. CIP^r: ciprofloxacin resistance phenotype; ND: not determined; S: susceptible; I: intermediate susceptibility; R: resistant.

Table S3: *Salmonella* Typhimurium								
**CIP[r]**	***gyrA***	***gyrB***	***parC***	***parE***	**PMQR**	**Efflux**	**MIC (mg/L)**	**References**
S-I	-	Glu466Asp	-	-	-	ND	0.06-0.25	[89, 126]
I	Asp87Asn	-	-	-	-	ND	0.125-0.25	[28, 34, 107, 112]
I	-	-	Thr57Ser	ND	-	ND	0.19	[34]
R	Asp87Asn	-	-	-	*aac(6')-Ib-cr*	ND	2-4	[124]
R	Asp87Asn	-	-	-	*aac(6')-Ib-cr, oqxA/B*	Yes	2-4	[153]
R	Asp87Asn	-	-	-	*aac(6')-Ib-cr, oqxA/B-qnrS1*	Yes	2- >32	[153]
R	Asp87Asn	-	-	-	*aac(6')-Ib-cr, qnrB*	Yes	2	[153]
I	Asp87Asn	-	Thr57Ser	-	*oqxA/B*	-	0.38	[32]
R	Asp87Asn	-	-	-	*oqxA/B*	Yes	4	[125, 153]
R	Asp87Gly	-	-	-	-	ND	1.12*	[108]
R	Asp87Gly	ND	-	ND	*aac(6')-Ib-cr*	ND	1	[134]
R	Asp87Tyr	-	-	-	*oqxAB*	ND	4	[125]
	Asp87Tyr	ND	-	ND	*qnrS1*	ND	0.25	[117]
I-R	Asp87Tyr	-	-	ND	-	ND	0.125-4	[34, 107, 117, 124]
R	Asp87Tyr	-	-	-	*aac(6')-Ib-cr*	ND	2-4	[124]
R	Asp87Tyr	-	-	-	*aac(6')-Ib-cr, qnrB*	ND	4	[124]
I	Ser83Tyr	-	Ser80Arg	-	-	ND	0.75	[32]
I	Ser83Tyr	-	-	-	-	No	0.12-1	[34, 89, 107, 112]
R	Ser83Tyr	ND	-	ND	*aac(6')-Ib-cr, qnrS1*	ND	8	[131]
I	Ser83Tyr	-	-	-	*oqxA/B*	ND	0.19	[32]
I	Ser83Phe	-	-	-	ND	ND	0.125-0.25	[107, 117]
R	Ser83Phe	-	-	-	*qnrS1*	ND	1.12*	[108]
R	Ser83Phe	-	-	-	*oqxA/B*	Yes	4	[125, 153]
I	-	-	-	Met438Ile	*qnrS1*	ND	0.38	[108]
I-R	Ser83Phe Asp87Asn	-	The57Ser Ser80Ile	ND	-	ND	0.19- >32	[34]
R	Ser83Phe Asp87Asn	-	The57Ser Ser80Arg	-	*aac(6')-Ib-cr*	ND	4	[124]
I	Ser83Phe Glu133Gly	-	-	ND	-	ND	0.125	[34]
R	Ser83Phe Asp87Asn	-	Ser80Arg	ND	-	ND	4-12	[34, 124, 125]
R	Ser83Phe Asp87Asn	-	Ser80Arg	-	*aac(6')-Ib-cr*	ND	4- >8	[124, 125]
R	Ser83Phe Asp87Asn		Ser80Arg	-	*qepA*	ND	>8	[125]
R	Ser83Phe Asp87Asn		Ser80Arg		*aac(6')-Ib-cr, oqxAB*		>8	[125]
R	Ser83Phe Asp87Gly	-	Ser80Arg	-	-	ND	4	[124]
R	Ser83Phe Asp87Gly Ala119Ser	ND	-	ND	-	Yes	512	[39]

Table S3: Mutations in the QRDRs of *Salmonella* Typhimurium. CLSI guidelines [58] were followed to ensure a uniform classification of isolates showing ciprofloxacin resistance. According to these guidelines, resistant isolates have an MIC of 1–2 mg/L, isolates with intermediate susceptibility have

an MIC of 0.12–0.5 mg/L, and susceptible isolates have an MIC of ≤ 0.06 mg/L. While, *S.* Typhimurium isolates have been reported that do not have QRDR mutations but carry PMQR elements, only isolates that have QRDR mutations are discussed in this review. *MIC is given as the geometrical mean value [108]. CIP^r: ciprofloxacin resistance phenotype; ND: not determined; S: susceptible; I: intermediate susceptibility; R: resistant.

**Table S4:** ***Salmonella*** **Hadar**							
**CIP^r**	***gyrA***	***parC***	***parE***	**PMQR**	**Efflux**	**MIC (mg/L)**	**References**
S-I	Asp87Tyr	-	-	-	ND	0.047-0.125	[108, 133]
I	Asp87Tyr	Thr57Ser	-	-	ND	0.25	[28, 134]
I	Asp87Asn	-	-	-	ND	0.094-0.125	[34, 133]
I	Asp87Asn	-	ND	-	Yes	0.75	[106]
I	Asp87Asn	Thr57Ser	-	-	ND	0.125-0.25	[28, 107]
I	Asp87Asn	Thr57Ser Ser80Ile	ND	-	ND	0.19	[34]
I	Asp87Asn	Thr57Ser	ND	*qnrD1*	ND	29mm (ZOI)	[34]
I	Ser83Phe	Thr57Ser	ND	-	ND	0.25	[34]
R	Ser83Phe Asp87Asn	Ser80Ile	-	-	ND	8	[133]
I	Ser83Phe Asp87Asn	Thr57Ser	ND	-	ND	0.38	[34]
I	Ser83Tyr	-	-	*qnrD2*	ND	0.75	[133]
S	Ser83Tyr	-	-	-	ND	0.064-0.125	[133]
R	Asp87Tyr	Arg96Ser Pro98Lys		-	ND	1.12*	[108]

Table S4: Mutations in the QRDR of *Salmonella* Hadar. CLSI guidelines [58] were followed to ensure a uniform classification of isolates showing ciprofloxacin resistance. According to these guidelines, resistant isolates have an MIC of 1–2 mg/L, isolates with an intermediate susceptibility have an MIC of 0.12–0.5 mg/L, and susceptible isolates have an MIC of ≤ 0.06 mg/L. None of the listed isolates carried a mutation in the *gyrB* gene. Similarly, no mutations were reported for the *parE* gene, but this was not studied in all isolates (as indicated by ND). *MIC is given as the geometrical mean value [108]. CIP^r: ciprofloxacin resistance phenotype; ZOI: zone of inhibition; ND: not determined; S: susceptible; I: intermediate susceptibility; R: resistant.

Table S5: *Salmonella* Kentucky							
CIP^r	*gyrA*	*parC*	*parE*	PMQR	Efflux	MIC (mg/L)	References
I	Ser83Tyr	-	-	-	ND	0.125	[133]
‡I	Ser83Phe	-			No	0.125-0.5	[41]
I-R	Ser83Phe	Ser80Ile	-	-	ND	0.19-8	[133]
R	Ser83Phe	Ser80Ile	-	-	ND	6 - >32	[133]
I	Asp87Asn	Thr57Ser	ND	-	ND	0.19	[34]
	Asp87Gly	-	ND	-	ND	0.047	[117]
R	Ser83Phe	Thr57Ser Ser80Ile	ND	-	ND	8-32	[34]
R	Ser83Phe Asp87Gly	Ser80Ile	-	-	No	6-8	[133]
‡R	Ser83Phe Asp87Tyr	Ser80Ile	-	-	Yes	4-8	[41, 133]
‡R	Ser83Phe Asp87Gly	Ser80Ile	-	-	Yes	3-8	[41, 133]
R	Ser83Phe Asp87Gly	Thr57Ser Ser80Ile	-	-	ND	9 -14 mm (ZOI)	[30, 34]
R	Ser83Phe Asp87Tyr	Thr57Ser Ser80Ile	-	-	ND	6 - ≥ 8	[34, 148]
R	Ser83Phe Asp87Asn	Thr57Ser Ser80Ile	ND	-	ND	8 - >32	[34]
‡R	Ser83Phe Asp87Asn	Ser80Ile	-	*aac(6')-Ib-cr*	ND	≥ 4	[144]
‡R	Ser83Phe Asp87Asn	Ser80Ile			Yes	8-16	[41]
R	Ser83Phe Asp87Tyr	Thr57Ser Ser80Ile	ND	*qnrB1*	ND	32	[34]
R	Ser83Phe Asp87Asn	Thr57Ser Ser80Ile	ND	*qnrD*	ND	24	[34]
S	-	Thr57Ser	ND	*qnrD*	ND	0.064	[34]
R	-	Thr57Ser	-	*qnrS1*	ND	8	[145]
I	-	Thr57Ser	ND	*qnrB1/ qnrD*	ND	0.25	[34]

Table S5: Mutations in the QRDRs of *Salmonella* Kentucky. CLSI guidelines [58] were followed to ensure a uniform classification of isolates showing ciprofloxacin resistance. According to these guidelines, resistant isolates have an MIC of 1–2 mg/L, isolates with intermediate susceptibility have an MIC of 0.12–0.5 mg/L, and susceptible isolates have an MIC of ≤ 0.06 mg/L. None of the listed isolates was found to have a mutation in the *gyrB* gene. Similarly, no mutations were reported for the *parE* gene, but this was not studied in all isolates (as indicated by ND). CIP^r: ciprofloxacin resistance phenotype; ZOI: zone of inhibition; ND: not determined; S: susceptible; I: intermediate susceptibility; [R]: resistant. ‡*Salmonella* Kentucky isolates were confirmed to be ST198 [41, 144].

Table S6: *Salmonella* Indiana						
**CIP^r**	**GyrA**	**GyrB**	**ParC**	**PMQR**	**MIC (mg/L)**	**References**
S-R	-	-	Thr57Ser	-	0.06-1	[107]
R	Ser83Leu	-	Ser80Ile	*aac(6')-Ib-cr*	4	[150]
R	Ser83Leu Asp87Asn	-	Thr57Ser Ser80Arg	*aac(6')-Ib-cr, oqxA*	32	[149]
R	Ser83Ile Asp87Asn	-	-	*aac(6')-Ib-cr*	2	[150]
I	Ser83Tyr	-	-	*qnrA*	0.25	[149]
R	Ser83Tyr	-	Ser80Ile	*aac(6')-Ib-cr*	2	[150]
R	Ser83Phe Asp87Gly		Thr57Ser	*oqxA*	128	[149]
R	Ser83Phe Asp87Gly	-	Thr57Ser Ser80Arg	*oqxAB*	8-64	[151]
R	Ser83Phe Asp87Asn	-	Thr57Ser Ser80Arg	*oqxAB*	32	[151]
R	Ser83Phe	-	Thr57Ser Ser80Arg	*oqxAB/ qepA*	32	[151]
R	Ser83Phe Asp87Asn	-	-	*aac(6')-Ib-cr/ oqxAB*	*>32	[153]
R	Ser83Phe Asp87Asn	-	Ser80Arg	*aac(6')-Ib-cr, oqxAB, qnrB*	*>32	[153]
R	Ser83Phe Asp87Asn	-	Thr57Ser Ser80Arg	*aac(6')-Ib-cr*	32-64	[151, 152]
R	Ser83Phe Asp87Asn	-	Thr57Ser Ser80Arg	-	8-16	[151]
R	Ser83Phe Asp87Asn	-	Thr57Ser Ser80Arg	*aac(6')-Ib-cr, oqxAB*	64-128	[151, 152]
R	Ser83Phe Asp87Asn	-	Thr57Ser Ser80Arg	*aac(6')-Ib-cr, oqxA, qnrA*	64	[149]
R	Ser83Phe Asp87Gly	-	Thr57Ser Ser80Arg	*aac(6')-Ib-cr oqxA, qnrA*	64	[149]
R	Ser83Phe Asp87Gly	-	Thr57Ser Ser80Arg	*aac(6')-Ib-cr, oqxAB*	64-128	[151, 152]
R	Ser83Phe Asp87Gly	-	Thr57Ser Ser80Arg	*aac(6')-Ib-cr*	16	[149]
R	Ser83Phe Asp87Gly	-	Thr57Ser Ser80Arg	-	8-128	[151, 152]
R	Ser83Phe Asp87Asn	-	Cys72Gly Ser80Arg	*aac(6')-Ib-cr, oqxAB*	*>32	[153]

Table S6: Mutations in the QRDRs of *Salmonella* Indiana. CLSI guidelines [58] were followed to ensure a uniform classification of isolates showing ciprofloxacin resistance. According to these guidelines, resistant isolates have an MIC of 1–2 mg/L, isolates with intermediate susceptibility have an MIC of 0.12–0.5 mg/L, and susceptible isolates have an MIC of ≤ 0.06 mg/L. None of the listed isolates was found to have a mutation in the *gyrB* and *parE* genes. *Efflux inhibition resulted in a reduction of the MIC to >8 mg/L [153]; efflux was not studied for the other isolates. CIP^r: ciprofloxacin resistance phenotype; S: susceptible; I: intermediate susceptibility, R: resistant.

**Table S7: *Salmonella* Infantis**						
**CIP^r**	***gyrA***	***gyrB***	***parC***	**PMQR**	**MIC (mg/L)**	**References**
I-R	Ser83Tyr	-	Thr57Ser	-	0.25-1	[28, 32, 34, 114]
I	-	-	Thr57Ser	*qnrA1*	0.75	[34]
I	Ser83Tyr	Leu470Met	Thr57Ser	-	0.5	[28]
R	Ser83Tyr	-	Ser80Arg	-	2	[155]
R	Ser83Tyr	-	-	-	1-2	[155]
S	Ser83Phe	-	-	-	0.064	[112]
I	Ser83Leu	-	-	-	< 1	[91]
I	Asp87Tyr	-	-	-	< 1	[91, 156]
I-R	Asp87Asn	-	-	-	0.25-1	[114, 156]
I	Asp87Gly	-	Thr57Ser	-	0.25	[34]
I	Leu41Pro<br>Asp87Tyr	-	-	-	< 1	[91]

Table S7: Mutations in the QRDRs of *Salmonella* Infantis. CLSI guidelines [58] were followed to ensure a uniform classification of isolates showing ciprofloxacin resistance. According to these guidelines, resistant isolates have an MIC of 1–2 mg/L, isolates with intermediate susceptibility have an MIC of 0.12–0.5 mg/L, and susceptible isolates have an MIC of ≤ 0.06 mg/L. The isolates reported by Hamidian et al. [91] showed MIC to CIP ≤ 1 mg/L, and, according to the updated CLSI guidelines, were thus classified as having intermediate susceptibility. None of the listed isolates was found to have a mutation in the *parE* gene. Efflux was not studied for any of the listed isolates. CIP^r: ciprofloxacin resistance phenotype; S: susceptible; I: intermediate susceptibility; R: resistant.

Table S8: *Salmonella* Derby							
**CIP^r**	***gyrA***	***gyrB***	***parC***	**PMQR**	**Efflux**	**MIC (mg/L)**	**References**
R	Asp87Gly	ND	-	*aac(6')-Ib-cr*	ND	1	[134]
I	Asp87Tyr	-	Thr57Ser	-	ND	0.12	[34]
I	Asp87Asn	-	Thr57Ser	-	ND	28mm (ZOI)	[34]
I	Ser83Phe	-	Thr57Ser	-	ND	0.19	[34]
R	Ser83Ile		ND	*oqxAB*	Yes	4	[153]
R	Ser83Tyr	-	-	*aac(6')-Ib-cr, oqxAB, qnrS2*	No	4	[153]
R	Ser83Tyr	-	ND	*oqxAB, qnrS8, qnrB*	Yes	2	[153]
R	Ser83Tyr	-	ND	*oqxAB, qnrS1, qnrB*	Yes	>32	[153]
R	Ser83Tyr	-		*qnrS1*	Yes	2	[153]
R	Asn78His	-	-	*aac(6')-Ib-cr, oqxAB, qnrS2*	No	2	[153]
I	-	-	Thr57Ser	*qnrB5*	ND	0.38	[34]

Table S8: Mutations in the QRDRs of *Salmonella* Derby. CLSI guidelines [58] were followed to ensure a uniform classification of isolates showing ciprofloxacin resistance. According to these guidelines, resistant isolates have an MIC of 1–2 mg/L, isolates with intermediate susceptibility have an MIC of 0.12–0.5 mg/L, and susceptible isolates have an MIC of ≤ 0.06 mg/L. None of the isolates was found to have a mutation in the *parE* gene. CIP^r: ciprofloxacin resistance phenotype; ZOI: zone of inhibition; ND: not determined; S: susceptible; I: intermediate susceptibility; R: resistant.

Table S9: *Salmonella* Newport						
**CIP^r**	***gyrA***	***gyrB***	***parC***	**PMQR**	**MIC (mg/L)**	**References**
I	Asp87Gly	-	Thr57Ser	-	0.125	[28, 34]
S-R			Thr57Ser	-	0.06-1	[107]
R	Asp87Asn	-	-	-	1.12*	[108, 112]
R	Asp87Asn	-	Arg96Ser Pro98Thr	*qnrS1*	1.12*	[108]
I	Ser83Phe	-	Thr57Ser	-	0.19-0.25	[28, 34]
I-R	Ser83Tyr	-	Thr57Ser	-	0.25 -2	[28]
R	Ser83Tyr	-	Thr57Ser	*qnrS1/qnrS3*	4	[28]
I	Asp87Tyr	ND	Thr57Ser	-	0.125	[134]
I	-	-	Thr57Ser	*qnrS1*	0.25	[34]
I	Ser83Phe Asp87Asn	-	Thr57Ser	-	0.25	[34]

Table S9: Mutations in the QRDRs of *Salmonella* Newport. CLSI guidelines [58] were followed to ensure a uniform classification of isolates showing ciprofloxacin resistance. According to these guidelines, resistant isolates have an MIC of 1–2 mg/L, isolates with an intermediate susceptibility have an MIC of 0.12–0.5 mg/L, and susceptible isolates have an MIC of ≤ 0.06 mg/L. Partially resistant and completely resistant isolates of *S.* Newport show equal prevalence for mutations of residues 83 or 87 and no preference for substitution by a specific residue. However, the Thr57Ser mutation is prevalent in parC. None of the isolates was found to have a mutation in the *parE* gene. Efflux was not studied for any of the listed isolates. *MIC is given as the geometrical mean value [108]. CIP^r: ciprofloxacin resistance phenotype; ND: not determined; S: susceptible; I: intermediate susceptibility; R: resistant; ND: not determined.

Table S10: *Salmonella* Virchow						
**CIP^r**	***gyrA***	***gyrB***	***parC***	**PMQR**	**MIC (mg/L)**	**References**
I	Asp87Tyr	-	-	-	0.094	[133]
I	Asp87Tyr	-	-	*qnrB2*	0.25	[133]
I	Asp87Tyr	-	-	*qnrS1*	0.5	[133]
I	Asp87Tyr	-	-	-	0.094-0.75	[28, 34, 134]
I	Ser83Phe	-	-	-	0.25 – 0.5	[28, 30, 34, 134]
R	Ser83Phe			*qnrS1*	2	[166]
I	Asp87Tyr	-	-	-	0.094-0.125	[133]
I	Ser83Leu Asp87Asn	-	-	-	0.125	[28]
R	Ser83Phe Asp87Tyr	-	-	-	4	[119]

Table S10: Mutations in the QRDRs of *Salmonella* Virchow. CLSI guidelines [58] were followed to ensure a uniform classification of isolates showing ciprofloxacin resistance. According to these guidelines, resistant isolates have an MIC of 1–2 mg/L, isolates with intermediate susceptibility have an MIC of 0.12–0.5 mg/L, and susceptible isolates have an MIC of ≤ 0.06 mg/L. None of the isolates was found to have a mutation in the *parE* gene. Efflux was not studied for any of the listed isolates. The most prevalent mutation, Ser83Phe, was identified in isolates from Europe [28, 34], Africa [30] and Asia [134, 166]. CIP^r: ciprofloxacin resistance phenotype; S: susceptible; I: intermediate susceptibility; R: resistant.
